# The combined effect of exposures to vapours, gases, dusts, fumes and tobacco smoke on current asthma

**DOI:** 10.1111/crj.13512

**Published:** 2022-06-10

**Authors:** Hanna Hisinger‐Mölkänen, Hannu Kankaanranta, Tari Haahtela, Anssi Sovijärvi, Leena Tuomisto, Heidi Andersén, Ari Lindqvist, Helena Backman, Arnulf Langhammer, Eva Rönmark, Pinja Ilmarinen, Paula Pallasaho, Päivi Piirilä

**Affiliations:** ^1^ University of Helsinki Helsinki Finland; ^2^ Department of Respiratory Medicine Seinäjoki Central Hospital Seinäjoki Finland; ^3^ Faculty of Medicine and Health Technology Tampere University Tampere Finland; ^4^ Krefting Research Centre, Institute of Medicine University of Gothenburg Gothenburg Sweden; ^5^ Skin and Allergy Hospital, Helsinki University Hospital University of Helsinki Helsinki Finland; ^6^ Unit of Clinical Physiology, HUS Medical Imaging Center Helsinki University Central Hospital Helsinki Finland; ^7^ Thoracic Oncology Unit, Tema Cancer Karolinska University Hospital Stockholm Sweden; ^8^ Clinical Research Unit of Pulmonary Diseases Helsinki University Hospital Helsinki Finland; ^9^ Department of Public Health and Clinical Medicine, Section for Sustainable Health/the OLIN Unit Umeå University Umeå Sweden; ^10^ HUNT Research Centre, Department of Public Health and Nursing, NTNU Norwegian University of Science and Technology Levanger Norway; ^11^ Levanger Hospital Nord‐Trøndelag Hospital Trust Levanger Norway; ^12^ Welfare and Health Sector City of Espoo Finland

**Keywords:** asthma, ETS, exposure, smoking, symptoms, tobacco smoke, VGDF

## Abstract

Smoking, exposure to environmental tobacco smoke (ETS) and occupational exposure to vapours, gases, dusts or fumes (VGDF) increase asthma symptoms. The impact of combined exposure is less well established. We aimed to evaluate the risk of combined exposure to smoking, ETS and VGDF on the prevalence of current asthma and asthma‐related symptoms with a postal survey among a random population of 16,000 adults, aged 20–69 years (response rate 51.5%). The 836 responders with physician‐diagnosed asthma were included in the analysis. Of them, 81.9% had current asthma defined as physician‐diagnosed asthma with current asthma medication use or reported symptoms. There was a consistently increasing trend in the prevalence of current asthma by increased exposure. The highest prevalence of multiple symptoms was in smokers with VGDF exposure (92.1%) compared to the unexposed (73.9%, *p* = 0.001). In logistic regression analysis, combined exposure to several exposures increased the risk in all analysed symptoms (*p* = 0.002–0.007). In conclusion, smoking and exposure to ETS or VGDF increased the prevalence of current asthma and multiple symptoms. The combined exposure carried the highest risk. Preventive strategies are called for to mitigate exposure to tobacco smoke and VGDF.

## Graphical abstract

The prevalence of current asthma and asthma‐related symptoms was increased in adult asthmatics by exposure to tobacco smoke and vapours, gases, dusts or fumes (VGDF). Combined exposure increased the prevalence further. These results highlight the importance of prevention of airborne occupational exposures and smoking cessation in asthma treatment.

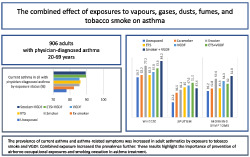

## 1 INTRODUCTION

Asthma is a heterogeneous disease by nature, and several factors are recognized to affect disease prognosis, control and symptom burden.^1^ Environmental factors play a role in asthma control,^2^ and indoor and outdoor pollution are risk factors both for asthma onset and worsening of asthma symptoms.^3^, ^4^, ^5^

The effect of smoking has been actively studied in asthma. According to several reports, smoking asthmatics are prone to have accelerated loss of lung function and worsened asthma control.^4^, ^6^, ^7^ A previous Finnish study reported increasing asthma risk in occasional to moderate smokers.^8^ Furthermore, smoking has been reported to have a dose‐dependent association with bronchial hyperresponsiveness in an adult Finnish population.^9^ The role of smoking in developing occupational asthma has also been of interest but results are inconsistent and the relationship seems to be complex. These controversial findings are suggested to be at least partly explained by ‘the healthy smoker effect’, meaning that asthmatics tend to be non‐smokers if they do not tolerate cigarette smoke.^8^, ^10^

Reporting multiple asthma symptoms has been associated with decreased lung function and increased bronchial hyperresponsiveness reflecting uncontrolled and more difficult asthma.^11^ Multiple factors like adherence to medication, comorbidities and exercise habits are known to affect asthma control, but it is also well established that respiratory symptoms may be caused by occupational factors. In a previous Finnish study, both occupational exposure and asthma diagnosis at adult age were associated with work‐related asthma symptoms.^12^, ^13^, ^14^, ^15^

Furthermore, occupational exposures are well known to cause occupational allergen‐induced or irritant induced asthma.^16^ Despite the possibility that an occupational specific allergen exposure or strong irritant exposure might be removed from workplaces or respiratory protection could be used, there may still be occupational vapours, gases, dusts or fumes (VGDF) which may cause respiratory symptoms to the workers. In addition, combined effect to tobacco smoke and occupational VGDF has been reported to have an additive effect on the risk in COPD and it has been suggested to increase the risk of asthma and COPD coexistence (ACO) in adult‐onset asthma but, to our best knowledge, the effect of combined exposure to tobacco smoke and VGDF is less studied on asthma symptoms.^17^, ^18^

Therefore, firstly, we aimed to test a hypothesis that combined exposure to tobacco smoke and VGDF would have an increasing effect on the prevalence of current asthma in all responders with physician‐diagnosed asthma. Secondly, we aimed to investigate if the prevalence of asthma‐related symptoms would be increased by combined exposure to tobacco smoke and VGDF in adults with current asthma.

## 2 MATERIAL AND METHODS

### 2.1 Characteristics of data

This study is a part of a FinEsS (Finland–Estonia–Sweden) study which began in 1996. In 2016, a cross‐sectional random sample of totally 16,000 20–69‐year‐olds, 8000 from both Helsinki and Western Finland areas, was collected corresponding to the Finnish population in 10‐year age cohorts, according to the gender and age distribution of the regions. Those invited were sent a FinEsS respiratory questionnaire, and up to two reminders were utilized. In total, 8199 responded. Those not reporting their smoking habits, exposure to VGDF or environmental tobacco smoke (ETS) were excluded from the analysis. A more detailed description of the study methods and questionnaire is reported elsewhere.^19^ A non‐responder phone interview study was planned but permission by the local ethical committee was denied.

### 2.2 Questions and definitions

The complete Finnish FinEsS 2016 questionnaire is available elsewhere.^20^

The key study variables were defined as follows.

*Physician‐diagnosed asthma*. ‘Have you been diagnosed by a doctor as having asthma?’

*Current asthma*. ‘Physician‐diagnosed asthma and positive answer to at least one of the following questions: (1) have you had attacks of breathlessness during the last 12 months, (2) have you had any wheeze during the last 12 months or (3) do you currently use asthma medication?’

*Exposure to ETS*. ‘Reporting exposure to ETS at home or work but no current smoking’

*Exposure to VGDF*. ‘Reporting heavy exposure to VGDF but no current smoking’

*Exposure to ETS and VGDF*. ‘Reporting exposure to both ETS and VGDF but no current smoking’

The definition of the common study variables can be found in the supporting information appendix.

In addition to asking about occupational exposure to VGDF, we evaluated occupational exposure based on reported professions by using the job‐exposure matrix (JEM). The reported job titles had been classified according to the International Standard Classification of Occupations (ISCO‐88) and each ISCO‐88 job category was separately weighted for biological dusts, mineral dusts and gases/fumes based on these individual components as presented by Mehta et al. High level of exposure was assessed according to Mehta et al. Low levels of exposures were assessed as probable or commonly known low exposure to biological and mineral dusts.^17^, ^21^

### 2.3 Statistical analyses

Statistical analyses were conducted with SPSS Statistics version 25. To compare categorical groups, a chi‐square test was utilized. Similarly, when comparing normally and non‐normally distributed continuous variables between two categorical variables, *t* test and Mann–Whitney test were utilized, respectively. A *P* value of <0.01 was considered statistically significant. Multivariable binary logistic regression was used to determine independent associations between covariates and different asthma symptoms, expressed as odds ratios (OR) and 95% confidence intervals (CI). The covariates were included in the model by potential confounding effect measured by individual statistical associations with the outcome variable as well as previous knowledge from the literature and clinical experience. Subjects with incomplete data on smoking habits and occupational exposure were excluded from the analyses. Sensitivity analyses were conducted by excluding responders with co‐existing COPD.

## 3 RESULTS

The 51.5% (*n* = 8199) responded after two reminders. Of the responders, 11.1% (906) reported physician‐diagnosed asthma and 9.0% (736) current asthma. Responders not reporting their smoking status or VGDF exposure status were excluded from the analysis and therefore 836 responders with physician‐diagnosed asthma and 685 with current asthma were included in this study. The demography of individuals with physician‐diagnosed asthma is seen in Table 1.

**TABLE 1:** Demographics of individuals with physician‐diagnosed asthma and current asthma

	All with physician‐diagnosed asthma *N* = 836	Men with physician‐diagnosed asthma *N* = 367	Women with physician‐diagnosed asthma *N* = 469	All with current asthma *N* = 685	
	median	median	median	median	*P*
Age (Q_1_‐Q_3_)	46.5 (32–61)	44 (32–59)	49 (33–61)	49 (33–61)	0.004
	**mean**	**mean**	**mean**	**mean**	
BMI (SD)	26.7 (5.3)	27.0 (5.3)	26.5 (5.5)	26.9 (5.4)	0.056
	**N (%)**	**N (%)**	**N (%)**	**N (%)**	
Male	367 (43.9)	N/A	N/A	282 (41.2)	N/A
COPD	79 (9.4)	43 (11.7)	36 (7.7)	76 (11.1)	0.047
Smoking					<0.001
Never	402 (48.1)	146 (39.8)	256 (54.6)	323 (47.2)	
Ex	247 (29.5)	122 (33.2)	125 (26.7)	202 (29.5)	
Current	187 (22.4)	99 (27.0)	88 (18.8)	160 (23.4)	
ETS	139 (16.6)	60 (16.3)	79 (16.8)	120 (17.5)	0.849
VGDF	243 (29.1)	128 (34.9)	115 (24.5)	208 (30.4)	<0.001
VGDF and ETS	76 (9.1)	37 (10.1)	39 (8.3)	68 (9.9)	0.013
VGDF and smoking	87 (10.4)	57 (15.5)	30 (6.4)	78 (11.4)	<0.001
Allergic rhinitis	480 (57.4)	208 (56.7)	272 (58.0)	402 (58.7)	0.702
Chronic rhinitis	474 (56.7)	204 (55.6)	270 (57.6)	415 (60.6)	0.566
Asthma medication use	611 (73.1)	244 (66.5)	367 (78.3)	611 (89.2)	<0.001
Family history of asthma	371 (44.4)	145 (39.5)	226 (48.2)	323 (47.2)	0.012
Exercise ≥2 times per week	576 (68.9)	230 (64.1)	346 (75.5)	479 (69.9)	<0.001

Abbreviations: BMI, body mass index; COPD, chronic obstructive pulmonary disease; ETS, environmental tobacco smoke; *p*, comparison between men and women with physician‐diagnosed asthmaVGDF, vapours, gases, dusts and fumes.

To find out if different exposures were associated with the prevalence of current asthma, we analysed the prevalence of current asthma in groups stratified by exposure history. Smoking and exposure to ETS, VGDF and combined exposures to VGDF with ETS or smoking increased the prevalence of current asthma, compared to the unexposed, and results were consistent in direction also in groups stratified by gender (Table 2).

**TABLE 2:** The prevalence (%) of current asthma among individuals ever been diagnosed with asthma by a physician, stratified by exposure status

Exposure status among individuals with physician‐diagnosed asthma	All current asthma *N* (%)	Men with current asthma *N* (%)	Women with current asthma *N* (%)
**Unexposed**	176 (77.9)	56 (75.7)	120 (78.9)
**Ex‐smoker**	209 (82.3)	97 (77.6)	112 (86.8)
	*P* = 0.227		
**Current smoker**	160 (85.6)	80 (80.8)	80 (90.9)
	*P* = 0.046		
**ETS**	120 (86.3)	51 (85.0)	69 (87.3)
	*P* = 0.045		
**VGDF**	208 (85.6)	101 (78.9)	107 (93.0)
	*P* = 0.030		
**VGDF and ETS**	68 (89.5)	32 (86.5)	36 (92.3)
	*P* = 0.027		
**VGDF and smoking**	76 (89.4)	51 (89.5)	27 (90.0)
	*P* = 0.017		

*Note*: *P* values are given between unexposed and those with tobacco smoke or/and VGDF exposure below the prevalence figures.

Abbreviations: ETS, environmental tobacco smoke; VGDF, vapours, gases, dusts and fumes.

When different asthma‐related symptoms were analysed in exposure status defined groups among all responders with current asthma, we saw an increasing trend in asthmatics reporting different symptoms and multiple symptoms by increasing level of exposure. Combined exposures to VGDF with ETS or smoking increased the occurrence of several asthma‐related symptoms. The highest prevalence of multiple symptoms was in smokers with VGDF exposure (92.1%) compared to the unexposed (73.9%, *p* = 0.001). (Table SE1).

To exclude the possibility of co‐existing COPD explaining the results we performed a sensitivity analysis in responders without co‐existing COPD. The results remained similar as we saw an increasing trend by increased exposure (Table SA2).

**FIGURE 1:**
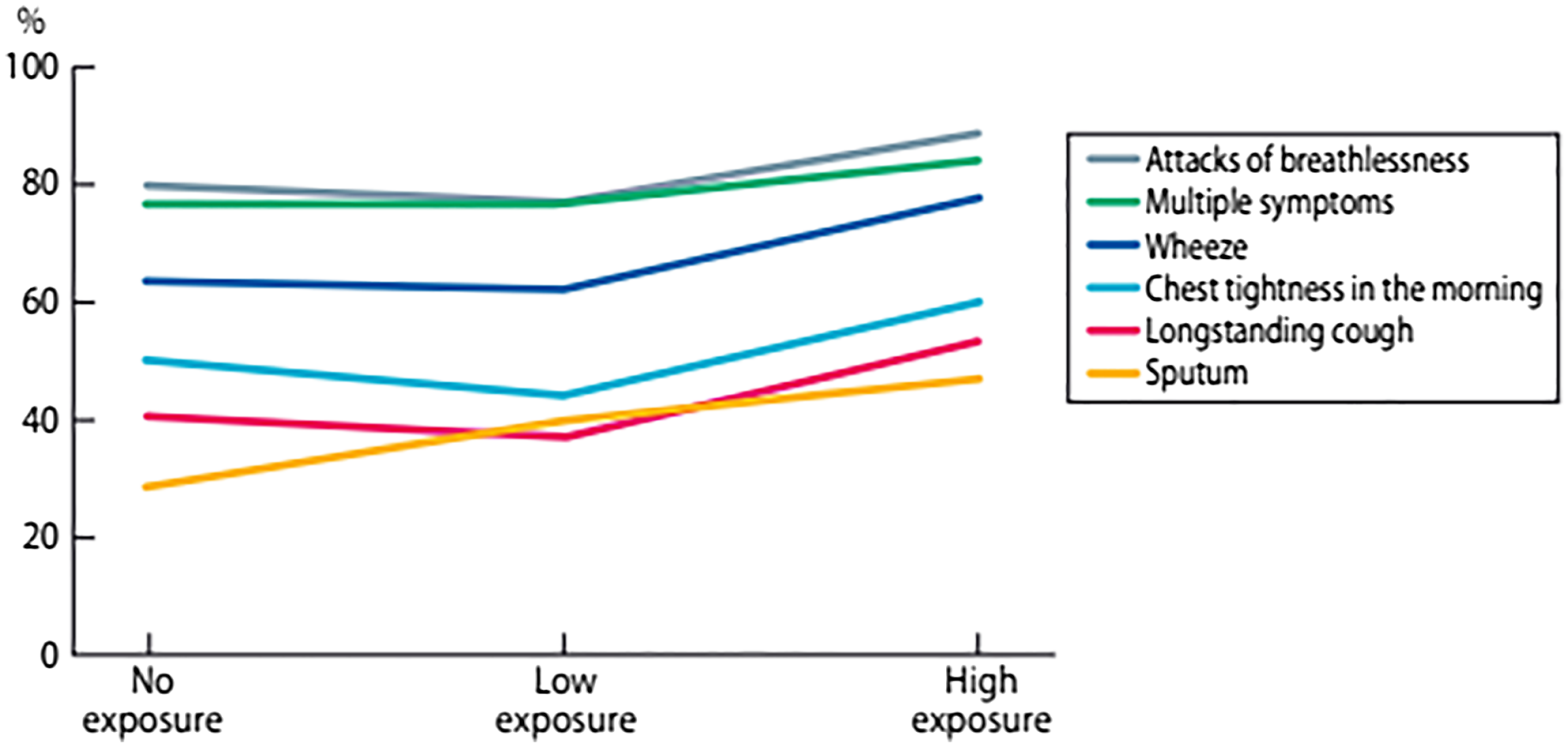
Prevalence (%) of different asthma‐related symptoms in responders with current asthma, stratified by occupational exposure based on professions according to the job‐exposure matrix (none = 0, low = 1–3, high = 4–6)

As exposure was also assessed by a JEM based on reported professions a level of VGDF exposure was constructed. There was a consistent trend for increasing symptoms with higher exposure (Figure 1).

To investigate the risk factors of longstanding cough, chest tightness in the morning and having multiple symptoms, we performed binary logistic regression analysis in responders with current asthma. Results are given in Table 3. Chronic rhinitis was a risk factor for all analysed symptoms. Female gender and BMI ≥ 30 were risk factors for multiple symptoms. As exposures were combined, increasing exposure increased the risk for all analysed symptoms. The greatest risk factor for having multiple symptoms was a cumulative exposure with three exposures.

**TABLE 3:** Risk factors of asthma‐related symptoms in current asthma by binary logistic regression analyses

	Cough		Chest tightness in the morning		Multiple symptoms (≥3)	
	OR (95% CI)	*p*	OR (95% CI)	*p*	OR (95% CI)	*p*
Female	1.51 (1.06–2.16)	0.022	1.35 (0.96–1.90)	0.088	1.75 (1.06–2.65)	**0.008**
Family history of asthma	1.09 (0.79–1.53)	0.594	1.04 (0.76–1.44)	0.792	1.13 (0.76–1.70)	0.562
Smoking						
Never	1		1		1	
Smoker	0.44 (0.26–0.77)	**0.003**	0.97 (0.58–1.62)	0.895	0.92 (0.47–1.80)	0.802
Ex‐smoker	0.49 (0.30–0.80)	**0.004**	0.52 (0.32–0.84)	**0.008**	0.55 (0.30–0.99)	0.045
BMI						
≤24.99	1		1		1	
25–29.99	0.83 (0.56–1.24)	0.371	1.49 (1.02–2.19)	0.041	1.67 (1.05–2.65)	0.031
≥30	1.36 (0.88–2.11)	0.162	1.74 (1.13–2.70)	0.011	2.17 (1.23–3.83)	**0.007**
Exercise <2 times per week	1.02 (0.72–1.52)	0.82	1.05 (0.74–1.53)	0.761	1.30 (0.81–2.09)	0.272
Chronic rhinitis	2.05 (1.44–2.91)	**<0.001**	1.67 (1.19–2.33)	**0.003**	2.175 (1.46–3.24)	**<0.001**
Allergic rhinitis	0.61 (0.43–0.87)	0.006	1.02 (0.72–1.43)	0.915	0.89 (0.58–1.36)	0.579
Age						
60–69	1.24 (0.72–2.12)	0.432	1.15 (0.67–1.96)	0.614	0.57 (0.30–1.08)	0.082
50–59	1.28 (0.72–2.28)	0.404	0.96 (0.55–1.67)	0.872	0.62 (0.32–1.23)	0.172
40–49	1.95 (1.10–3.47)	0.023	1.08 (0.63–1.88)	0.774	0.84 (0.41–1.72)	0.633
30–39	1.49 (0.85–2.64)	0.166	0.68 (0.40–1.14)	0.140	0.68 (0.35–1.31)	0.246
20–29	1		1		1	
Cumulative exposure						
No exposure	1		1		1	
1 exposure	1.35 (0.82–2.20)	0.236	1.67 (1.03–2.70)	0.039	1.14 (0.64–2.05)	0.657
2 exposures	2.38 (1.28–4.42)	**0.006**	2.11 (1.15–3.87)	0.016	2.11 (0.99–4.49)	0.054
3 exposures	3.43 (1.58–7.46)	**0.002**	2.89 (1.34–6.24)	**0.007**	7.12 (2.05–24.78)	**0.002**

*Note*: Cumulative exposure = 0–3 exposures of the following: being a smoker/ex‐smoker, environmental tobacco smoke (ETS) exposure and vapours, gases, dusts or fumes (VGDF) exp.

When logistic regression was performed with a similar model in responders without co‐existing COPD, the results were similar. The greatest risk factor for having multiple symptoms was the exposure to three exposures (Table SA3).

## 4 DISCUSSION

The prevalence of current asthma increased by increasing numbers of exposures in individuals with physician‐diagnosed asthma. Asthma‐related symptoms increased by exposures to tobacco smoke and VGDF in subjects with current asthma. Responders with combined exposure to both tobacco smoke and VGDF had the highest prevalence estimates for having multiple symptoms. Cumulative exposure increased the risk for analysed asthma‐related symptoms both in all with current asthma and as co‐existing COPD was excluded.

ETS increased the prevalence of several asthma‐related symptoms compared to the unexposed in our study. Our results are in line with previous findings. Previously, ETS has been reported to increase the prevalence of respiratory symptoms in never smokers^22^ and of more symptomatic asthma in asthmatics.^23^ Exposure to tobacco smoke is known to alter the immune system and therefore increase the risk for allergy and asthma.^24^ ETS exposure in childhood has also been linked to adult‐onset asthma.^25^ The prevalence of smoking has decreased in Finland in recent decades and therefore exposure to ETS will likewise probably decrease.^19^

Smoking is known to have harmful effects on asthma leading to both clinical and prognostic outcomes.^4^ Actively smoking asthmatics have a poorer prognosis both in lung function^6^ and in treatment outcome but still, the prevalence of smoking in asthma is close to that found in the general population.^4^ 22.4% of asthmatics reported active smoking in our study which is even higher than the smoking prevalence (21.2%) in the general population in our postal‐questionnaire study among all responders (unpublished data). Smoking may alter the response for asthma treatment and smoking asthmatics are more likely to have hospitalizations due to respiratory symptoms.^4^ Our study indicates that smoking increases the prevalence of current asthma and asthma‐related symptoms. Results were similar as co‐existing COPD was excluded.

Self‐reported exposure to VGDF is widely used in epidemiological questionnaire studies. When tested for performance against exposure likelihood by a JEM it was assessed as moderately sensitive and therefore a useful method in epidemiological studies.^26^ Occupational exposure to VGDF has been reported to increase the risk of asthma and rhinitis^5^ and to be associated with increased risk of COPD also in non‐smokers.^21^ An elevated risk for asthma and COPD exacerbations have been suggested by VGDF exposure but a recent study did not confirm this.^27^ In our study, occupational exposure to VGDF was associated with an increased prevalence of current asthma and several symptoms. When exposure status was categorized with JEM, our findings were similar.

The combined effect of tobacco smoke and occupational VGDF exposure has been less studied in asthma. Association to increased prevalence has been reported in COPD and asthma‐COPD overlap syndrome (ACO).^17^, ^18^ In our study, exposure to VGDF with ETS or smoking increased the prevalence of current asthma and asthma‐related symptoms and the highest prevalence figures for reporting multiple asthma symptoms were seen in these exposures. As exposure status was stratified by the number of different exposures (smoking/ex‐smoking, ETS and VGDF), an increasing trend of symptoms was seen in regression analysis. A possible mechanism behind this phenomenon could be thymic stromal lymphopoietin (TSLP) which is a regulator in asthma inflammation and increased by triggers such as pollutants. TSLP might be a mechanistical explanation for these increasing symptoms by increasing exposure.^28^

In occupational studies, the selection into job‐bias should be considered. Workers not in good health may avoid jobs with known exposure risk leading to workers in better health in exposure risky jobs.^29^ One could question if associations observed in our study are an underestimation due to possible healthy worker bias. When responders were stratified based on exposure by reported professions with JEM, we see slightly higher prevalence figures for longstanding cough and morning dyspnoea in the unexposed compared to those with low exposure. This might be explained by possible healthy worker bias.

The present study has several strengths. A large sample size, with a wide age span, reflects the general population well. Asthma diagnosed at all ages is included in our study sample. The study samples were randomly selected within 10‐year strata for each sex taking into account the overall gender and age distribution in the population. Our study population consists of two random cohorts collected from different areas in Finland, Helsinki being the capital in Southern Finland and the most urban area of our country and Seinäjoki–Vaasa a more country‐side area in Western Finland.

Participation rates have decreased in epidemiological studies in general. Therefore, we find our response rate moderate considering the general decline in response rates during the last decades.^30^ A telephone interview of a sample of non‐responders was planned but ethical permission was denied and therefore, we performed a non‐responder analysis based on information available. More detailed information on performed analysis is reported previously.^19^

The present study has also limitations. The study is mainly descriptive and based on self‐completed postal‐questionnaire. One could question the reliability of asthma diagnoses or mislabelling of COPD as asthma. However, as asthma is diagnosed with objective lung function testing to get special reimbursement for asthma medication costs in Finland, asthma diagnoses in this study are reliable. Symptoms of other disorders might be mislabelled as asthma symptoms, a problem to consider both in clinical practice and research. The study sample is not sufficient to address possible gender differences. Another limitation in questionnaire‐based studies may be that results are dependent on participants judgement. Additionally, exposure categories are not totally mutually exclusive and the questions do not have a grading system in FinEsS‐questionnaire (dichotomous yes or no answers). However, the increasing trend with increasing exposure is found as exposures were analysed in separate groups or in a cumulative manner in regression analyses and therefore the trend seems to be reliable. Thus, we believe that these considerations are not critical regarding the main results and the association of combined exposures on asthma‐related symptoms should be further studied in larger asthma populations.

## 5 CONCLUSIONS

The prevalence of current asthma and asthma‐related symptoms was increased in adult asthmatics by exposure to tobacco smoke and VGDF. Combined exposure to tobacco smoke and VGDF increased the prevalence further. Our results indicate the importance of the prevention of occupational airborne exposures and smoking cessation in asthma treatment.

## CONFLICT OF INTEREST

Päivi Piirilä reports financial support from Nordforsk Foundation. Leena Tuomisto reports payments for lectures from GlaxoSmithKline, AstraZeneca and Boehringer‐Ingelheim. Helena Backman reports payments for scientific presentations from AstraZeneca and Boehringer Ingelheim. Hannu Kankaanranta reports personal fees from AstraZeneca, Boehringer Ingelheim, Chiesi Pharma AB, GlaxoSmithKline, MSD, Orion Pharma, Novartis, Sanofi Genzyme and fees for lectures from AstraZeneca, Orion Pharma and Mundipharma. Arnulf Langhammer reports honoraria for lectures from AstraZeneca and Boehringer Ingelheim. Tari Haahtela reports lecturing fees from GSK, Mundipharma, Orion Pharma and Sanofi. Pinja Ilmarinen reports lecture fees from GlaxoSmithKline, Novartis, Mundipharma and AstraZeneca. Pinja Ilmarinen is an employee of GlaxoSmithKline. Hanna Hisinger‐Mölkänen has been an employee of GlaxoSmithKline and is an employee of Orion Pharma. The other authors have no competing interests.

## AUTHOR CONTRIBUTIONS

HH‐M, PPi and PPa designed the study and wrote the report. HH‐M performed the statistical analyses with help from PI. PPi, PPa, AL, AS and HH‐M contributed to the FinEsS Helsinki sample, population sample and questionnaires. HK, PI, HA and LT contributed to the FinEsS Western sample. TH, ER, AL and HB contributed to interpretation of the data and made critical revisions of the manuscript. All authors read and approved the final version of the publication and gave consent to publication.

## ETHICS STATEMENT

Informed written consent was obtained from all responders. The Ethics Committee of the Department of Medicine of Helsinki University Central Hospital gave approval (200/13/03/00/15). The study was conducted in accordance with the Declaration of Helsinki.

## Supporting information


**Table E1.** Prevalence (%) of asthma‐related symptoms in individuals with current asthma according to exposure history. P‐values are given between unexposed and those who are exposed to one or more of the risk factors.
ETS = environmental tobacco smoke, VGDF = vapours, gases, dusts and fumes
Table E2 Prevalence of asthma symptoms in all responders with current asthma and no co‐existing COPD according to exposure history. P‐values are given between unexposed and those who are exposed to one or more of the risk factors.
Table E3.Risk factors of asthma symptoms in binary multivariable logistic regression in current asthma and no co‐existing COPD.

## Data Availability

The data are available within this article and in the supporting information.
