# Supplementary material for: The combined effect of exposures to vapours, gases, dusts, fumes and tobacco smoke on current asthma
Source: Clin Respir J. 2022 Jun 10;16(6):467–74. doi: 10.1111/crj.13512 (PMC9366564; doi:10.1111/crj.13512)
Supplement: Supplementary file 1 — Table E1. Prevalence (%) of asthma‐related symptoms in individuals with current asthma according to exposure history. P‐values are given between unexposed and those who are exposed to one or more of the risk factors. ETS = environmental tobacco smoke, VGDF = vapours, gases, dusts and fumes Table E2 Prevalence of asthma symptoms in all responders with current asthma and no co‐existing COPD according to exposure history. P‐values are given between unexposed and those who are exposed to one or more of the risk factors. Table E3.Risk factors of asthma symptoms in binary multivariable logistic regression in current asthma and no co‐existing COPD. [file CRJ-16-467-s001.docx]

**APPENDIX**

The common study variables were defined as follows:

*Asthma medication use.* ‘Do you currently use asthma medication (regularly or as needed)’?

*Allergic rhinitis.* *‘*Have you been diagnosed by a physician as having allergic rhinitis caused by pollen (birch, grass, mug wort) or ‘Have you been diagnosed by a physician as having allergic rhinitis caused by other allergens than pollens (e.g., cat or dog)’?

*Chronic rhinitis.* ‘Have you had longstanding nasal congestion’ or ‘Have you had longstanding rhinitis’?

*Family history of asthma.* ‘Have any of your parents, brothers or sisters now or previously had asthma’?

*COPD.* ‘Have you been diagnosed by a physician as having chronic bronchitis, chronic obstructive pulmonary disease (COPD) or emphysema’?

*Occupational exposure to VGDF*. ‘Does your working environment have now or had before a lot of dusts, gases or fumes’?

*Living in rural area in childhood. ‘*Did you live in the countryside (not in a city or suburb) during the first 5 years of your life’?

*Living on a farm in childhood*. ‘Did you live on a farm during the first 5 years of your life’?

*Exercise per week.* ‘Exercise in your free time: How often do you exercise at least 30 minutes so that you are slightly short of breath and get sweaty’?

*Wheeze*. ‘Have you had wheezing or whistling in your chest at any time during the last 12 months’?

*Longstanding cough.* ‘Have you had longstanding cough during the last 12 months?’

*Attacks of breathlessness.* ‘Do you have now, or have you had asthma symptoms during the last 12 months (breathlessness with or without cough or wheezing)’?

*Sputum production.* ‘Do you bring up phlegm on most days during periods of at least three successive months?’

*Asthmatic wheeze.* Reporting *wheeze* and positive responses to ‘Have you been at all breathless when the wheezing sound was present’? and ‘Have you had this wheezing or whistling when you did not have a cold’?

*Breathlessness in cold.* ‘Do you usually have dyspnea or severe cough in cold weather’?

*Breathlessness in exercise. ‘*Do you usually have dyspnea or severe cough during exercise’?

*Chest tightness in the morning.* ‘Have you awakened with a feeling of tightness in your chest during the past 12 months’?

*Breathlessness mMRC≥2.* ‘Do you have to walk slower than other people of your age because of breathlessness’?

*Multiple symptoms*. Reporting positive answers to three or more questions of the nine asthma symptoms inquired

**Table E1. Prevalence (%) of asthma-related symptoms in individuals with current asthma according to exposure history. P-values are given between unexposed and those who are exposed to one or more of the risk factors.**

|  | Unexposed  N (%) | Ex-smoker  N (%) | Current smoker  N (%) | ETS  N (%) | VGDF  N (%) | ETS+VGDF  N (%) | Smoker + VGDF  N (%) |
| --- | --- | --- | --- | --- | --- | --- | --- |
| Wheeze | **105 (59.7)** | 132 (63.5)  p=0.446 | **122 (77.2)**  **P=0.001** | 76 (63.3)  P=0.525 | 141 (67.8)  P=0.099 | 50 (73.5)  P=0.044 | **64 (84.2)**  **p<0.001** |
| Longstanding cough | 66 (37.5) | 81 (38.9)  P=0.772 | 60 (38.0)  P=0.929 | 54 (45.0)  P=0.198 | **107 (51.4)**  **P=0.006** | 33 (48.5)  P=0.116 | 36 (47.4)  P=0.144 |
| Attacks of breathlessness | 139 (79.0) | 161 (77.4)  P=0.711 | 127 (80.4)  P=0.751 | 96 (80.0)  P=0.831 | 166 (79.8)  P=0.841 | 54 (79.4)  P=0.940 | 65 (85.5)  P=0.225 |
| Sputum production | 39 (22.2) | **76 (36.5)**  **P=0.002** | **65 (41.1)**  **p<0.001** | **54 (45.0)**  **p<0.001** | **99 (47.6)**  **p<0.001** | **35 (51.5)**  **p<0.001** | **33 (43.4)**  **P=0.001** |
| Asthmatic wheeze | 51 (29.0) | 72 (34.6)  P=0.239 | 60 (38.0)  P=0.082 | 40 (33.3)  P=0.426 | 71 (34.1)  P=0.280 | 28 (41.2)  P=0.068 | 33 (43.4)  P=0.026 |
| Breathlessness in cold | 108 (61.4) | 118 (56.7)  P=0.842 | 85 (53.8)  P=0.163 | 75 (62.5)  P=0.844 | 127 (61.1)  P=0.951 | 41 (60.3)  P=0.878 | 45 (59.2)  P=0.749 |
| Breathlessness in exercise | 104 (59.1) | 125 (60.1)  P=0.359 | 97 (61.4)  P=0.428 | 76 (63.3)  P=0.464 | 132 (63.5)  P=0.381 | 48 (70.6)  P=0.097 | 46 (60.5)  P=0.832 |
| Chest tightness in the morning | 69 (39.2) | 89 (42.8)  P=0.478 | **91 (57.6)**  **P=0.001** | 52 (43.3)  P=0.479 | **113 (54.3)**  **P=0.003** | 34 (50.0)  P=0.127 | **47 (61.8)**  **P=0.001** |
| Breathlessness  mMRC≥2 | 40 (22.7) | **74 (35.6)**  **P=0.006** | 55 (34.8)  P=0.015 | **47 (39.2)**  **P=0.002** | **83 (39.9)**  **p<0.001** | **32 (47.1)**  **p<0.001** | **30 (39.5)**  **P=0.007** |
| Multiple symptoms | 130 (73.9) | 158 (76.0)  P=0.637 | **132 (83.5)**  P=0.032 | 98 (81.7)  P=0.118 | 170 (81.7)  P=0.063 | 59 (86.8)  P=0.031 | **70 (92.1%)**  **P=0.001** |

ETS= environmental tobacco smoke, VGDF= vapours, gases, dusts and fumes

**Table E2** **Prevalence of asthma symptoms in all responders with current asthma and no co-existing COPD according to exposure history. P-values are given between unexposed and those who are exposed to one or more of the risk factors.**

|  | Unexposed  N=173  N (%) | Ex-smoker  N=181  N (%) | Smoker  N=129  N (%) | ETS  N=99  N (%) | VGDF  N=176  N (%) | ETS+VGDF  N=52  N (%) | Smoker + VGDF  N=57  N (%) |
| --- | --- | --- | --- | --- | --- | --- | --- |
| Wheeze | 102 (59.0) | 113 (62.4)  P=0.504 | **96 (75.0)**  **P=0.004** | 61 (61.6)  P=0.668 | 117 (66.5)  P=0.147 | 38 (73.1)  P=0.066 | **47 (83.9)**  **P=0.001** |
| Longstanding cough | 64 (37.0) | 72 (39.8)  P=0.591 | 43 (33.6)  P=0.543 | 46 (46.5)  P=0.126 | **92 (52.3)**  **P=0.004** | 27 (51.9)  P=0.055 | 23 (41.1) p=0.586 |
| Attacks of breathlessness | 137 (79.2) | 140 (77.3)  P=0.675 | 99 (77.3)  P=0.701 | 80 (80.8)  P=0.750 | 140 (79.5)  P=0.935 | 42 (80.8)  P=0.805 | 46 (82.1)  P=0.633 |
| Sputum production | 39 (22.5) | **67 (37.0)**  **P=0.003** | 45 (35.2)  P=0.016 | **48 (48.5)**  **p<0.001** | **86 (48.9)**  **p<0.001** | **30 (57.7)**  **p<0.001** | 18 (32.1)  P=0.150 |
| Asthmatic wheeze | 51 (29.5) | 61 (33.7)  P=0.394 | 46 (35.9)  P=0.237 | 32 (32.3)  P=0.625 | 58 (33.0)  P=0.484 | 21 (40.4)  P=0.140 | 23 (41.4)  P=0.108 |
| Breathlessness in cold | 106 (61.3) | 104 (57.5)  P=0.466 | 66 (51.6)  P=0.093 | 61 (61.6)  P=0.955 | 107 (60.8)  P=0.927 | 32 (61.5)  P=0.972 | 31 (55.4)  P=0.434 |
| Breathlessness in exercise | 103 (59.5) | 104 (57.5)  P=0.692 | 78 (60.9)  P=0.245 | 59 (59.6)  P=0.992 | 105 (59.7)  P=0.982 | 35 (67.3)  P=0.314 | 33 (58.9)  P=0.936 |
| Chest tightness in the morning | 68 (39.3) | 80 (44.2)  P=0.352 | 69 (53.9)  P=0.012 | 46 (46.5)  P=0.251 | **100 (56.8)**  **P=0.001** | 30 (57.7)  P=0.019 | **33 (58.9)**  **P=0.010** |
| Breathlessness mMRC≥2 | 38 (22.0) | 58 (32.0)  P=0.033 | 35 (27.3)  P=0.283 | 31 (31.3)  P=0.089 | **61 (34.7)**  **P=0.009** | 18 (34.6)  P=0.065 | 16 (28.6)  P=0.313 |
| Multiple symptoms | 127 (73.4) | 136 (75.1)  P=0.710 | 102 (79.7)  P=0.208 | 80 (80.8)  P=0.169 | 143 (81.3)  P=0.081 | 45 (86.5)  P=0.051 | 50 (89.3)  P=0.014 |

**TABLE E3.**

**Risk factors of asthma symptoms in binary multivariable logistic regression in current asthma and no co-existing COPD.**

|  | Cough |  | Chest tightness in the morning |  | Multiple symptoms (≥3) |
| --- | --- | --- | --- | --- | --- |
|  |  |  |  |  |  |
| Female | **1.77 (1.21-2.60)** |  | **1.52 (1.06-2.19)** |  | **1.87 (1.22-2.87)** |
| Family history of asthma | 1.10 (0.77-1.57) |  | 1.00 (0.71-1.42) |  | 1.07 (0.70-1.61) |
| Smoking |  |  |  |  |  |
| Never | 1 |  | 1 |  | 1 |
| Smoker | 0.38 (0.21-0.68) |  | 0.77 (0.44-1.35) |  | 0.75 (0.38-1.51) |
| Ex-smoker | 0.56 (0.33-0.94) |  | 0.54 (0.33-0.90) |  | 0.58 (0.32-1.08) |
| BMI |  |  |  |  |  |
| ≤24.99 | 1 |  | 1 |  | 1 |
| 25-29.99 | 0.89 (0.59-1.36) |  | **1.52 (1.01-2.28)** |  | **1.65 (1.02-2.68)** |
| ≥30 | 1.46 (0.92-2.33) |  | **1.743 (1.10-2.76)** |  | **1.94 (1.08-3.45)** |
| Exercise <2 times per week | 0.97 (0.65-1.45) |  | 1.04 (0.71-1.54) |  | 1.21 (0.75-1.96) |
| Chronic rhinitis | **1.99 (1.37-2.89)** |  | **1.79 (1.25-2.55)** |  | **2.21 (1.46-3.34)** |
| Allergic rhinitis | 0.56 (0.38-0.81) |  | 0.97 (0.67-1.39) |  | 0.83 (0.54-1.30) |
| Age  60-69  50-59  40-49  30-39  20-29 | 1.40 (0.78-2.49)  **1.94 (1.06-3.53)**  1.28 (0.70-2.34)  1.05 (0.59-1.86)  1 |  | 1.05 (0.61-1.80)  0.94 (0.53-1.66)  0.91 (0.51-1.63)  0.57 (0.33-0.98)  1 |  | 0.54 (0.29-1.03)  0.58 (0.29-1.16)  0.76 (0.36-1.58)  0.58 (0.30-1.14)  1 |
| Cumulative exposure  No exposure  1 exposure  2 exposures  3 exposures | 1  1.37 (0.82-2.28)  **2.48 (1.29-4.76)**  **3.93 (1.64-9.38)** |  | 1  1.58 (0.95-2.61)  **2.31 (1.22-4.37)**  **4.78 (1.98-11.54)** |  | 1  1.08 (0.60-1.97)  **2.24 (1.03-4.90)**  **10.55 (2.13-52.25)** |
